# COPD patients with high blood eosinophil counts exhibit a lower rate of omicron infection and milder post‐infection symptoms

**DOI:** 10.1111/crj.13790

**Published:** 2024-05-30

**Authors:** Xueli Bai, Yanan Niu, Shuang Wei, Zhifan Zhu, Min Xu, Hu Liu, Xiansheng Liu, Ruiying Wang

**Affiliations:** ^1^ Third Hospital of Shanxi Medical University, Shanxi Bethune Hospital, Shanxi Academy of Medical Sciences, Tongji Shanxi Hospital Taiyuan China; ^2^ Tongji Hospital, Tongji Medical College Huazhong University of Science and Technology Wuhan China

**Keywords:** COPD, EOS, follow‐up, protective effect, SARS‐CoV‐2

## Abstract

**Background:**

The emergence of the severe acute respiratory syndrome coronavirus 2 (SARS‐CoV‐2) and its subsequent Omicron variant has raised concerns for chronic obstructive pulmonary disease (COPD) patients due to the potential risk of disruptions to healthcare services and unknown comorbidities between COPD and Omicron.

**Method:**

In this study, we conducted a follow‐up investigation of 315 COPD patients during the Omicron outbreak at Shanxi Bethune Hospital to understand the impact of the pandemic on this vulnerable population. Among all patients, 228 were infected with Omicron, of which 82 needed hospitalizations.

**Result:**

We found that COPD patients with high blood eosinophil (EOS) counts exhibited lower susceptibility to Omicron infection and were more likely to have milder symptoms that did not require hospitalization. Conversely, patients with low EOS counts showed higher rates of infection and hospitalization. Moreover, EOS count was positively correlated with T lymphocyte counts in hospitalized patients after Omicron infection, suggesting potential associations between EOS and specific immune responses in COPD patients during viral infections. Correlation analysis revealed a positive correlation between EOS count and lymphocyte and T‐cells, and a negative correlation between EOS count and age, neutrophil, and C‐reactive protein.

**Conclusion:**

Overall, our study contributes to the knowledge of COPD management during the COVID‐19 Omicron outbreak and emphasizes the importance of considering individual immune profiles to improve care for COPD patients in the face of the ongoing global health crisis.

## Graphical abstract

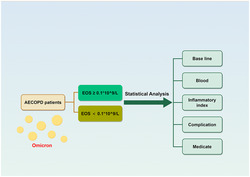

## 1 INTRODUCTION

Chronic obstructive pulmonary disease (COPD) is a chronic, progressive respiratory disease that results in limitations in lung airflow.^1^ Due to its high prevalence, increasing incidence, and significant economic costs, COPD has become a major global health concern. The World Health Organization predicted that COPD will become the third leading cause of death worldwide by 2030.^2^ COPD is also recognized as a complex multi‐component disease involving chronic systemic inflammation, often accompanied by other coexisting conditions known as comorbidities.^3^, ^4^, ^5^, ^6^ Until now, effective therapies that can modify COPD outcomes are still lacking. Blood eosinophil (EOS) count has been proposed as a biomarker for guiding corticosteroid therapy during COPD exacerbations and identifying patients who may benefit from treatment regimens.^7^, ^8^ However, the clinical application and mechanisms of EOS in COPD patients still require a deeper understanding.

The emergence of severe acute respiratory syndrome coronavirus 2 (SARS‐CoV‐2) has led to a global pandemic that poses a significant threat to human lives.^9^ Coronavirus disease 2019 (COVID‐19) can present with a range of symptoms, from asymptomatic or mild cases to severe pneumonia‐like symptoms. The Omicron variant has raised concerns due to its high number of mutations and potential impact on transmissibility and vaccine effectiveness, and it has quickly become the dominant strain, surpassing the Delta variant.^10^ COPD patients face additional stressors during the pandemic as they fear the risk of contracting COVID‐19 and the potential effects of the pandemic on essential societal functions and health services.^11^ Moreover, the unknown comorbidities between COPD and the Omicron variant further contribute to their concerns.^12^, ^13^, ^14^ It is essential to understand the implications for COPD patients to provide optimal care and support during this challenging time.

In this study, we conducted a follow‐up on COPD patients during the Omicron outbreak at Shanxi Bethune Hospital between 5 October 2022 and 1 February 2023. We collected and analyzed comprehensive data on the overall Omicron infection in COPD patients, including infection rate, hospitalization rate, cure rate, mortality rate, and other relevant statistics. To investigate the role of EOS counts in COPD patients, we compared clinical differences between uninfected and infected cases, as well as hospitalized and non‐hospitalized patients, by grouping them based on EOS counts (EOSC: ≥0.1 × 10^9^/L and <0.1 × 10^9^/L). Our study contributes valuable insights into COPD management during the COVID‐19 Omicron outbreak.

## 2 METHODS AND MATERIALS

### 2.1 Patients

This trial was designed to investigate the implications for COPD patients during the Omicron outbreak. A total of 714 COPD patients were hospitalized in Shanxi Bethune Hospital during the 1 year from 1 September 2021 to 1 September 2022. We conducted a follow‐up study on COPD patients during the Omicron variant outbreak in 2022 (it was validated by RNA sequencing). There were 347 patients followed up, among whom 32 had unfortunately passed away. The patients were excluded based on the following criteria: (1) a combination of asthma, allergic diseases, hypereosinophilia, parasitic infections, blood system‐related diseases, and other conditions known to affect EOS; (2) complicated with other obvious respiratory diseases, such as pulmonary tuberculosis, bronchiectasis, and lung cancer, (3) use of long‐term oral corticosteroid or had used oral or intravenous corticosteroids before the admission. Finally, 315 patients were included in this study, and 228 COPD patients were infected with Omicron. The present study was approved by the ethics committee of Shanxi Bethune Hospital ([2022] S022), and all participants provided written informed consent.

### 2.2 Clinical indicators collection

Based on the data collected from 315 patients during their COPD treatment, we recorded their age, body mass index (BMI), blood indices, other diseases present in addition to COPD, and medications utilized for COPD treatment. A total of 17 blood indicators were summarized, including oxygen partial pressure (PO_2_), partial pressure of carbon dioxide (PCO_2_), white blood cell (WBC), neutrophil percentage (NEUP), neutrophil count (NEUC), lymphocyte percentage (LYP), lymphocyte count (LYC), eosinophil percentage (EOSP), eosinophil count (EOSC), hemoglobin (HB), platelet (PLT), C‐reactive protein (CRP), fibrinogen (FIB), albumin (ALB), D_dimer, and procalcitonin (PCT). Besides COPD, we also observed the occurrence of 10 other diseases in the patients, including concurrent pulmonary nodules, chronic pulmonary heart disease (CPHD), hypertension, diabetes, coronary heart disease (CHD), cardiac arrhythmias, cerebral infarction, mental disease, gastroesophageal reflux (GR), and Type II respiratory failure (Type II RF). For daily medication, we analyzed the use of the four specific inhaled medications, namely, budesonide/glycopyrronium/formoterol (BGF), tiotropium, salmeterol/fluticasone (SF), and budesonide/formoterol (BFF). After infection with Omicron, 82 COPD patients required hospitalization, and we conducted a follow‐up study to re‐measure blood indices. Patients with a positive nucleic acid of SARS‐CoV‐2 were required to meet the following inclusion criteria for admission to the hospital: (1) experiencing shortness of breath with a respiratory rate (RR) of ≥30 times/min; (2) exhibiting resting oxygen saturation of ≤93%; (3) having an oxygen partial pressure in arterial blood/inhaled oxygen concentration ratio of ≤300 mmHg; (4) displaying lung imaging indicating that lesions have progressed by more than 50% within 24 to 48 h; (5) showing progressive aggravation of clinical symptoms, including (a) respiratory failure requiring mechanical ventilation; (b) shock; (c) experiencing combined organ failures with requirement of intensive care unit monitoring and treatment.

### 2.3 EOS grouping

We compared clinical differences between uninfected and infected cases, as well as hospitalized and non‐hospitalized patients, by grouping them based on EOS counts (EOSC: ≥0.1 × 10^9^/L and <0.1 × 10^9^/L). The high EOS group is EOSC ≥0.1 × 10^9^/L, and the low EOS group is EOSC <0.1 × 10^9^/L.^15^, ^16^

### 2.4 Statistical analysis

*χ* ^2^ test and Fischer's test were analyzed using SPSS 17.0 (SPSS Institute, Chicago, IL, USA). Statistical significance was set at *p* < 0.05.

## 3 RESULTS

### 3.1 Follow‐up of COPD patients during the omicron outbreak

**FIGURE 1:**
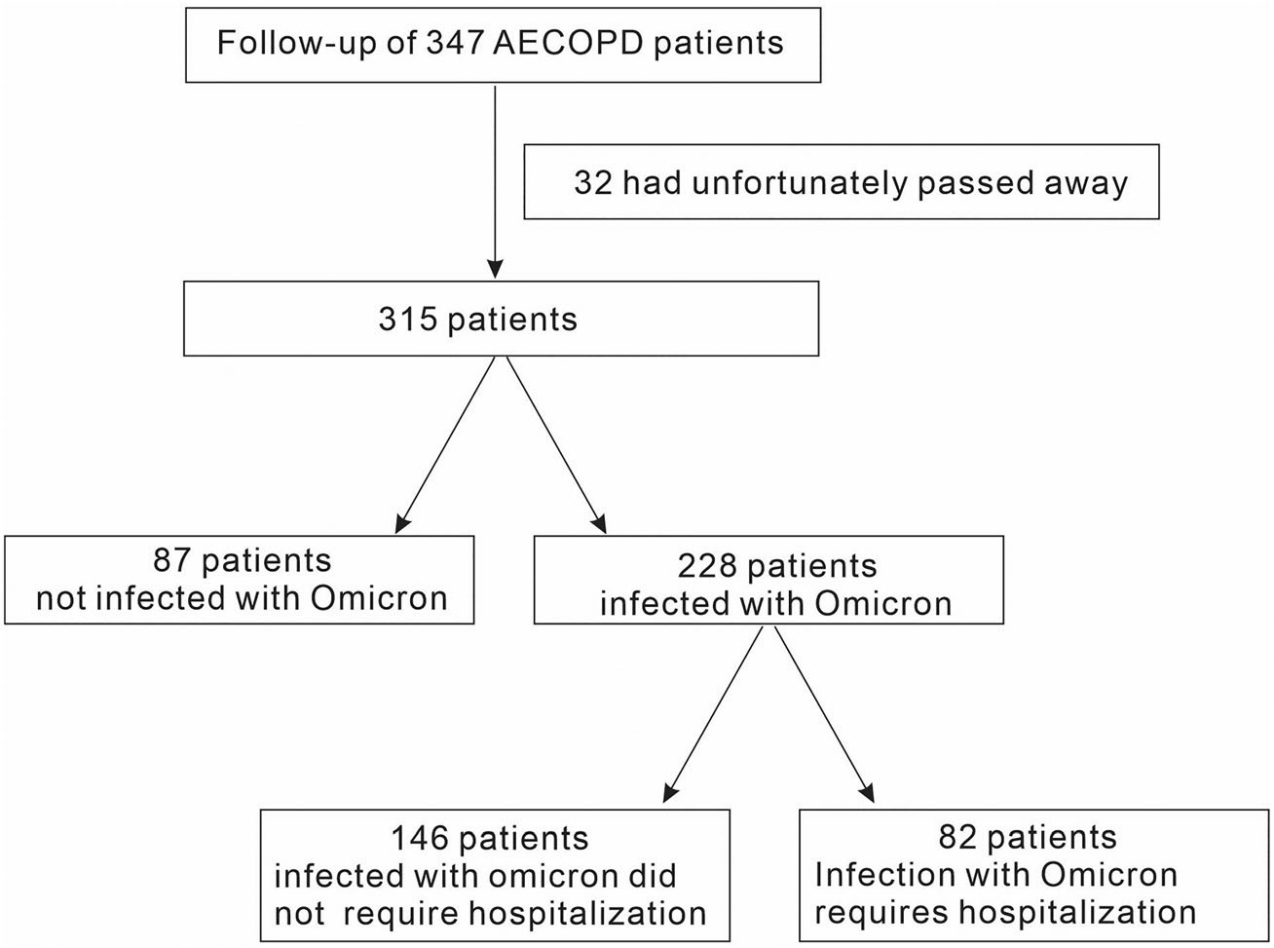
Flow chart of patient composition.

A total of 714 COPD patients were hospitalized in Shanxi Bethune Hospital during the 1 year from 1 September 2021 to 1 September, 2022. During the 2022 Omicron variant outbreak (it was validated by RNA sequencing), we investigated a cohort of COPD patients hospitalized in the previous year. A total of 347 patients were followed up, among whom 32 had unfortunately passed away. Finally, 315 patients were included in this study (Figure 1), comprising 253 (80.32%) males and 62 (19.68%) females, with a mean age of 70.14 years and a mean BMI of 23.64. Based on the data collected from 315 patients during their COPD treatment, we analyzed the blood indices, other diseases present in addition to COPD, and medications utilized for COPD treatment. A total of 16 blood indicators were summarized, including EOSC less than 0.1 × 10^9^/L in 158 (50.16%) patients and EOSC greater than or equal to 0.1 × 10^9^/L in 157 (49.84%) patients, LYC less than 2 × 10^9^/L in 132 (41.9%) patients, and LYC greater than or equal to 2 × 10^9^/L in 183 (58.1%) patients, as well as PO_2_ less than 60 mmHg in 147 (46.67%) patients and PO_2_ greater than or equal to 60 mmHg in 168 (53.33%) cases. Besides COPD, we also observed 20 other coexisting diseases in these patients. For instance, concurrent pulmonary nodules were found in 47 (14.92%) patients, CPHD in 125 (39.68%) patients, concurrent hypertension in 99 (31.43%) patients, and concurrent diabetes mellitus in 21 (6.67%) patients. For daily medication, we analyzed the use of each of the four specific medications with 129 (40.95%) patients utilizing BGF, 40 (12.7%) patients using tiotropium, 40 (12.7%) patients using SF, and 71 (22.54%) patients employing BFF. During the Omicron outbreak, 228 (72.38%) of 315 COPD patients were infected with the Omicron virus, and 82 (35.96%) of them were hospitalized for treatment of more severe symptoms (Figure 1).

### 3.2 COPD patients with high EOS counts are more likely to be protected from Omicron

**FIGURE 2:**
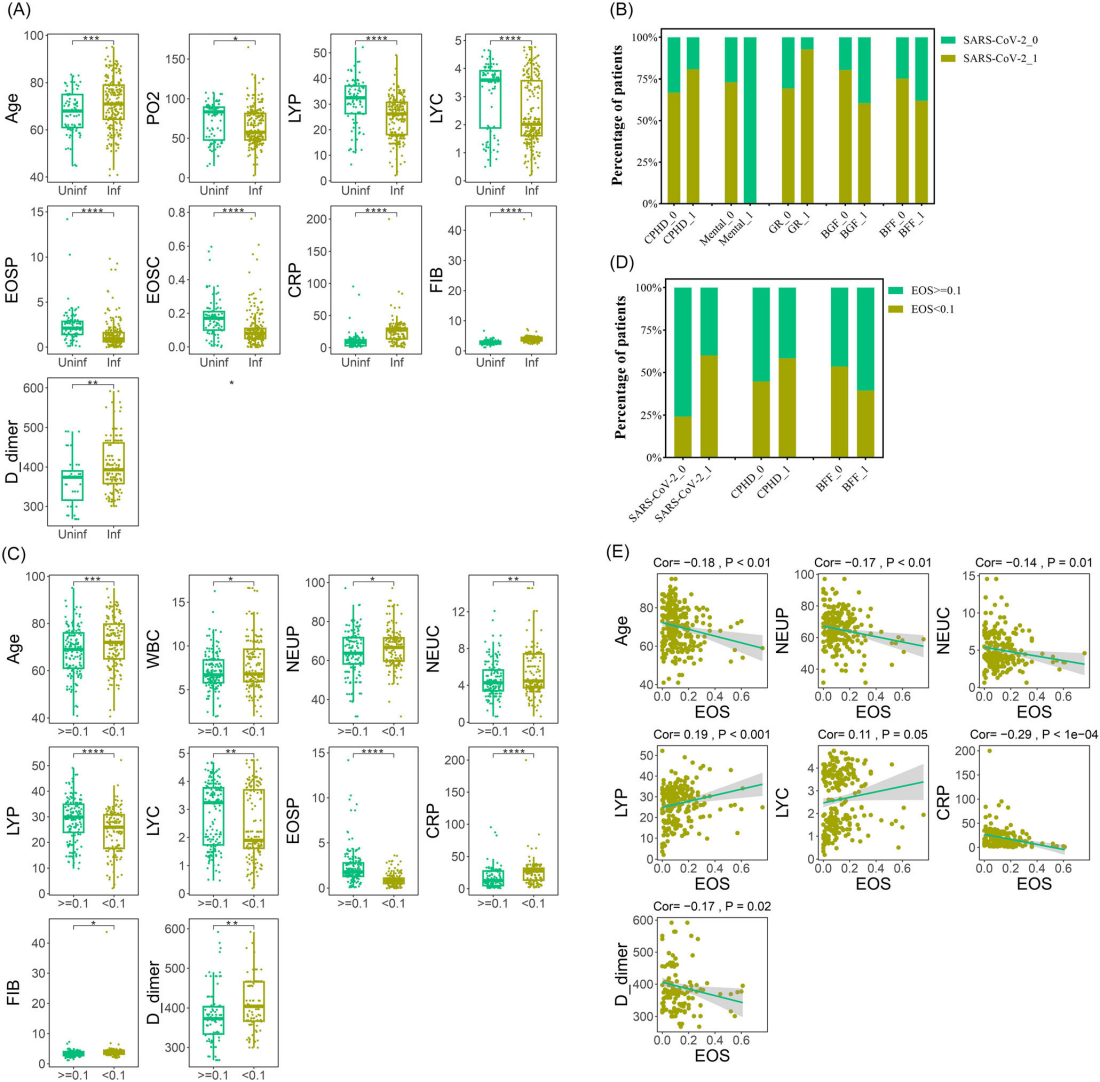
Clinical indicators of significant differences between chronic obstructive pulmonary disease (COPD) patients not infected with Omicron and infected with Omicron. (A) Blood indicators of significant differences between uninfected and infected groups; (B) co‐existing diseases and daily medications of significant differences between uninfected and infected groups; (C) blood indicators of significant differences between uninfected and infected patients grouped by eosinophil (EOS) count (EOSC); (D) co‐existing diseases and daily medications of significant differences between uninfected and infected patients grouped by EOSC; (E) blood indicators with significant correlation with EOS in uninfected and infected groups.

By comparing the clinical data of 87 COPD patients not infected with Omicron and 228 COPD patients infected with Omicron, we observed several indicators of significant differences. The age of COPD patients infected with Omicron was higher than those who were not infected (71.26 vs. 67.21, *p* < 0.01), and levels of CRP (24.7 vs. 10.79, *p* < 0.0001), FIB (4.2 vs. 2.89, *p* < 0.0001), and D_dimer (402.09 vs. 364.5, *p* < 0.01) were also higher in COPD patients infected with Omicron. Moreover, PO_2_ (72.31 vs. 64.91, *p* = 0.013), LY (percentage: 30.79% vs. 25.06%, *p* < 0.0001; count: 3.09 vs. 2.43, *p* < 0.0001), EOS (percentage: 2.41% vs. 1.36%, *p* < 0.0001; count: 0.17 vs. 0.1, *p* < 0.0001) were higher in COPD patients who were not infected with Omicron. For the coexisting diseases, we found that COPD patients with CPHD (80.8% vs. 66.84%, *p* < 0.01) and GR (92.68% vs. 69.34%, *p* < 0.01) had a higher infection rate with Omicron compared with COPD patients without mental disease (0% vs. 73.08%, *p* = 0.02). There were only three COPD patients with mental disease in total, which may have affected the accuracy of the results. On daily medication, we found that COPD patients treated with BGF (60.47% vs. 80.33%, *p* < 0.001) or BFF (61.97% vs. 75.1%, *p* = 0.03) were more likely to be not infected with Omicron (Tables 1 and 2, Figure 2A,B).

**TABLE 1:** *T*‐test results between chronic obstructive pulmonary disease patients un‐infected and infected with Omicron.

Type	SARS‐CoV‐2					EOS				
	Uninfected		Infected		*p*	EOS < 0.1		EOS ≥ 0.1		*p*
	Total	Mean	Total	Mean		Total	Mean	Total	Mean	
Age	87	67.21	227	71.26	0.001858	158	72.06	156	68.19	0.000905
BMI	12	22.47	85	23.80	0.242844	54	23.18	43	24.21	0.191975
Oxygen partial pressure	87	72.31	228	64.91	0.013037	158	65.63	157	68.30	0.317679
Partial pressure of carbon dioxide	87	44.69	228	45.88	0.32032	158	45.48	157	45.63	0.908387
White blood cell	87	7.45	228	7.53	0.841105	158	7.88	157	7.13	0.010689
Neutrophil percentage	87	65.21	228	65.15	0.970394	158	66.35	157	63.97	0.048841
Neutrophil count	87	5.09	228	5.00	0.804914	158	5.37	157	4.68	0.006405
Lymphocyte percentage	87	30.79	228	25.06	1.36E‐06	158	24.46	157	28.84	4.47E‐06
Lymphocyte count	87	3.09	228	2.43	1.01E‐05	158	2.41	157	2.82	0.002626
Eosinophil percentage	87	2.41	228	1.36	9.92E‐06	158	0.93	157	2.38	3.31E‐15
Eosinophil count	87	0.17	228	0.10	5.39E‐07	NA	NA	NA	NA	NA
Hemoglobin	84	137.74	216	139.98	0.259322	149	137.78	151	140.91	0.110851
Platelet	84	220.63	216	199.74	0.104206	149	201.57	151	209.55	0.393518
C‐reactive protein	80	10.79	209	24.70	1.42E‐10	145	25.31	144	16.36	2.73E‐05
Fibrinogen	70	2.89	136	4.20	6.09E‐05	95	4.23	111	3.35	0.048634
Albumin	63	37.32	174	37.82	0.479304	121	37.05	116	38.36	0.066252
D_dimer	42	364.50	133	402.09	0.001572	90	406.59	85	378.75	0.006347
Procalcitonin	27	0.09	40	0.08	0.340571	43	0.07	24	0.10	0.15688

Abbreviations: BMI, body mass index; EOS, eosinophil.

**TABLE 2:** Chi‐square/Fisher's test between chronic obstructive pulmonary disease patients un‐infected and infected with Omicron.

Type	SARS‐CoV‐2					EOS				
	Positive		Negative		*p*	Positive		Negative		*p*
	Infected	Uninfected	Infected	Uninfected		EOS ≥ 0.1	EOS < 0.1	EOS ≥ 0.1	EOS < 0.1	
Gender	47 (75.81%)	15 (24.19%)	181 (71.54%)	72 (28.46%)	0.500865	37 (59.68%)	25 (40.32%)	120 (47.43%)	133 (52.57%)	0.083914
Nodules	38 (80.85%)	9 (19.15%)	190 (70.9%)	78 (29.1%)	0.159125	20 (42.55%)	27 (57.45%)	137 (51.12%)	131 (48.88%)	0.278638
Chronic Pulmonary heart disease	101 (80.8%)	24 (19.2%)	127 (66.84%)	63 (33.16%)	0.006714	52 (41.6%)	73 (58.4%)	105 (55.26%)	85 (44.74%)	0.017654
Hypertension	70 (70%)	30 (30%)	158 (73.49%)	57 (26.51%)	0.519206	48 (48%)	52 (52%)	109 (50.7%)	106 (49.3%)	0.655782
Diabetes	16 (72.73%)	6 (27.27%)	212 (72.35%)	81 (27.65%)	0.969951	12 (54.55%)	10 (45.45%)	145 (49.49%)	148 (50.51%)	0.647268
Coronary heart disease	19 (76%)	6 (24%)	209 (72.07%)	81 (27.93%)	0.673173	8 (32%)	17 (68%)	149 (51.38%)	141 (48.62%)	0.062964
Arrhythmology	22 (70.97%)	9 (29.03%)	206 (72.54%)	78 (27.46%)	0.852963	14 (45.16%)	17 (54.84%)	143 (50.35%)	141 (49.65%)	0.583109
Cerebral infarction	24 (80%)	6 (20%)	204 (71.58%)	81 (28.42%)	0.326472	13 (43.33%)	17 (56.67%)	144 (50.53%)	141 (49.47%)	0.453559
Mental disease	0 (0%)	3 (100%)	228 (73.08%)	84 (26.92%)	0.020542	3 (100%)	0 (0%)	154 (49.36%)	158 (50.64%)	0.243708
Gastroesophageal reflux	38 (92.68%)	3 (7.32%)	190 (69.34%)	84 (30.66%)	0.001824	18 (43.9%)	23 (56.1%)	139 (50.73%)	135 (49.27%)	0.414806
Type II respiratory failure	78 (76.47%)	24 (23.53%)	150 (70.42%)	63 (29.58%)	0.261268	44 (43.14%)	58 (56.86%)	113 (53.05%)	100 (46.95%)	0.099606
Budesonide/glycopyrronium/formoterol	78 (60.47%)	51 (39.53%)	147 (80.33%)	36 (19.67%)	0.000117	72 (55.81%)	57 (44.19%)	83 (45.36%)	100 (54.64%)	0.068829
Tiotropium	28 (70%)	12 (30%)	197 (72.43%)	75 (27.57%)	0.749322	24 (60%)	16 (40%)	131 (48.16%)	141 (51.84%)	0.162059
Salmeterol/fluticasone	31 (77.5%)	9 (22.5%)	194 (71.32%)	78 (28.68%)	0.416013	17 (42.5%)	23 (57.5%)	138 (50.74%)	134 (49.26%)	0.330729
Budesonide/formoterol	44 (61.97%)	27 (38.03%)	181 (75.1%)	60 (24.9%)	0.030108	43 (60.56%)	28 (39.44%)	112 (46.47%)	129 (53.53%)	0.036889
SARS‐CoV‐2	NA	NA	NA	NA	NA	91 (39.91%)	137 (60.09%)	66 (75.86%)	21 (24.14%)	1.16E‐08

Abbreviation: EOS, eosinophil.

Based on the finding that EOS count was higher in COPD patients not infected with Omicron, we conducted further analysis by grouping the data using absolute quantification of EOS count (EOSC <0.1 vs. EOSC ≥0.1), which allowed us to determine the clinical indicators associated with EOS count and their potential relevance to Omicron infection. The results showed that low EOSC was more likely to be associated with Omicron infection (60.09% vs. 24.14%, *p* < 0.0001). Additionally, we observed that age (72.06 vs. 68.19, *p* < 0.001), WBC (7.88 vs. 7.13, *p* = 0.01), NEU (66.35% vs. 63.97%, *p* = 0.049; count: 5.37 vs. 4.68, *p* < 0.01), CRP (25.31 vs. 16.36, *p* < 0.0001), FIB (4.23 vs. 3.35, *p* = 0.049), and D_dimer (406.59 vs. 378.75, *p* < 0.01) were higher in the low EOSC group while LY (28.84% vs. 24.46%. *p* < 0.0001; count: 2.82 vs. 2.41, *p* < 0.01) was higher in high EOSC group. For the coexisting diseases, we found that COPD patients with CPHD (58.4% vs. 44.74%, *p* = 0.02) were more prevalent in the low EOSC group. On daily medication, we found that COPD patients treated with BFF (60.56% vs. 46.47%, *p* = 0.04) were more prevalent in the high EOSC group. Furthermore, we performed the correlation analysis between the absolute quantification of EOSC and all other blood indices, which showed a positive correlation between EOSC and LY (percentage: Cor = 0.19, *p* < 0.001; count: Cor = 0.11, *p* = 0.05), and a negative correlation between EOSC and age (cor = −0.18, *p* < 0.01), NEU (percentage: Cor = −0.17, *p* < 0.01; count: Cor = −0.14, *p* = 0.01), CRP (Cor = −0.29, *p* < 0.0001), and D_dimer (Cor = −0.17, *p* = 0.02). In summary, our results indicate that COPD patients with high EOSC were more likely to be protected from Omicron infection. High EOSC was also associated with high LY levels while lower EOSC was correlated with higher age, NEU, CRP, and D_dimer levels in COPD patients (Tables 1 and 2, Figure 2C–E).

### 3.3 COPD patients with high EOSC experience milder symptoms after the Omicron infection

**FIGURE 3:**
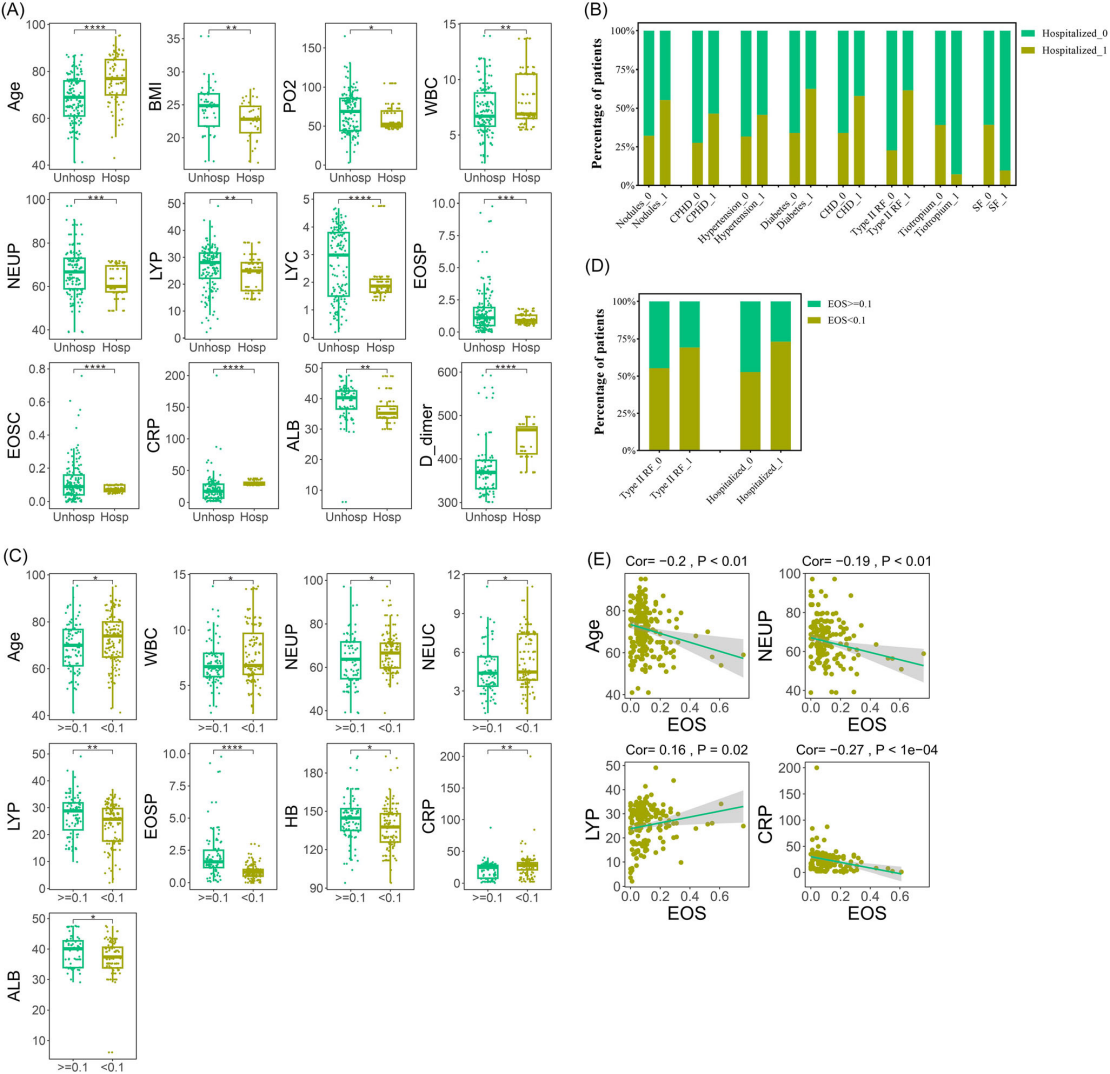
Clinical indicators of significant differences between chronic obstructive pulmonary disease (COPD) patients infected with Omicron who did not require hospitalization and required hospitalization. (A) Blood indicators that significantly differed between un‐hospitalized and hospitalized groups; (B) co‐existing diseases and daily medications significantly differed between un‐hospitalized and hospitalized groups; (C) blood indicators that significantly differed between un‐hospitalized and hospitalized patients grouped by eosinophil (EOS); (D) co‐existing diseases and daily medications significantly differed between un‐hospitalized and hospitalized patients grouped by eosinophil (EOS); (E) blood indicators correlated significantly with EOS in patients with un‐hospitalized and hospitalized.

Among the 228 COPD patients infected with Omicron, 146 patients exhibited mild symptoms and did not require hospitalization, while the remaining 82 cases had severe symptoms and needed hospitalization. To explore the clinical characteristics of these two groups (un‐hospitalization and hospitalization), we compared the clinical indicators between them. The results revealed that Age (76.4 vs. 68.41, *p* < 0.0001), WBC (8.12 vs. 7.19, *p* < 0.01), CRP (30.44 vs. 20.99, *p* < 0.0001), and D_dimer (441.66 vs. 380.47, *p* < 0.0001) were higher in the hospitalized patients. While PO2 (67.33 vs. 60.61, *p* = 0.02), NEU (Percentage: 66.75% vs. 62.31%, *p* < 0.001), LY (Percentage: 26.01% vs. 23.36%, *p* < 0.01; Count: 2.65 vs. 2.05, *p* < 0.0001), EOS (Percentage: 1.58% vs. 0.98%, *p* < 0. 001; Count: 0.12 vs. 0.07, *p* < 0.0001), and ALB (38.83 vs. 36.33, *p* = 0.02) were higher in patients who did not require hospitalization. Furthermore, we examined the impact of coexisting diseases on hospitalization rates. COPD patients with Nodules (55.26% vs. 32.11%, *p* < 0.01), CPHD (46.53% vs. 27.56%, *p* < 0.01), Hypertension (45.71% vs. 31.65%, *p* = 0.04), Diabetes (62.5% vs. 33.96%, *p* = 0.02), CHD (57.89% vs. 33.97%, *p* = 0.04) and Type II RF (61.54% vs. 22.67%, *p* < 0.0001) had higher hospitalization rates after infecting with Omicron. Additionally, patients who did not use Tiotropium (39.09% vs. 7.14%, *p* < 0.001) and SF (39.18% vs. 9.68%, *p* < 0.01) as part of their daily medication had higher hospitalization rates (Tables 3 and 4, Figure 3A,B).

**TABLE 3:** *T*‐test results between chronic obstructive pulmonary disease patients infected with Omicron without hospitalization and hospitalized.

Type	SARS‐CoV‐2					EOS				
	Un‐hospitalized		Hospitalized		*p*	EOS < 0.1		EOS ≥ 0.1		*p*
	Total	Mean	Total	Mean		Total	Mean	Total	Mean	
Age	146	68.41	81	76.40	2.51E‐08	137	72.58	90	69.26	0.021402
BMI	52	24.58	33	22.57	0.010391	52	23.27	33	24.63	0.086419
Oxygen partial pressure	146	67.33	82	60.61	0.017955	137	66.11	91	63.11	0.344349
Partial pressure of carbon dioxide	146	45.81	82	46.01	0.897138	137	45.64	91	46.25	0.707886
White blood cell	146	7.19	82	8.12	0.004606	137	7.82	91	7.09	0.015675
Neutrophil percentage	146	66.75	82	62.31	0.000252	137	66.40	91	63.27	0.020477
Neutrophil count	146	4.93	82	5.15	0.426399	137	5.27	91	4.60	0.011959
Lymphocyte percentage	146	26.01	82	23.36	0.008073	137	23.70	91	27.10	0.001043
Lymphocyte count	146	2.65	82	2.05	2.38E‐05	137	2.38	91	2.51	0.401084
Eosinophil percentage	146	1.58	82	0.98	0.000132	137	0.85	91	2.14	2.33E‐08
Eosinophil count	146	0.12	82	0.07	1.48E‐05	NA	NA	NA	NA	NA
Hemoglobin	140	140.11	76	139.74	0.886022	128	137.37	88	143.78	0.009638
Platelet	140	194.52	76	209.35	0.115908	128	200.04	88	199.31	0.9367
C‐reactive protein	127	20.99	82	30.44	8.60E‐06	127	27.64	82	20.15	0.001611
Fibrinogen	106	4.37	30	3.59	0.058637	81	4.47	55	3.81	0.204675
Albumin	104	38.83	70	36.33	0.003049	105	36.99	69	39.10	0.01983
D_dimer	86	380.47	47	441.66	3.17E‐09	81	407.93	52	393.00	0.215111
Procalcitonin	22	0.10	18	0.05	0.065071	33	0.07	7	0.12	0.33063

Abbreviations: BMI, body mass index; EOS, eosinophil.

**TABLE 4:** Results of *χ* ^2^/Fisher test between patients with chronic obstructive pulmonary disease infected with omicron without hospitalization and hospitalized.

Type	SARS‐CoV‐2					EOS				
	Positive		Negative		*p*	Positive		Negative		*p*
	Hospitalized	Un‐hospitalized	Hospitalized	Un‐hospitalized		EOS ≥ 0.1	EOS < 0.1	EOS ≥ 0.1	EOS < 0.1	
Gender	19 (40.43%)	28 (59.57%)	63 (34.81%)	118 (65.19%)	0.474489	23 (48.94%)	24 (51.06%)	68 (37.57%)	113 (62.43%)	0.15624
Nodules	21 (55.26%)	17 (44.74%)	61 (32.11%)	129 (67.89%)	0.006617	12 (31.58%)	26 (68.42%)	79 (41.58%)	111 (58.42%)	0.250518
Chronic pulmonary heart disease	47 (46.53%)	54 (53.47%)	35 (27.56%)	92 (72.44%)	0.003019	37 (36.63%)	64 (63.37%)	54 (42.52%)	73 (57.48%)	0.367317
Hypertension	32 (45.71%)	38 (54.29%)	50 (31.65%)	108 (68.35%)	0.04117	26 (37.14%)	44 (62.86%)	65 (41.14%)	93 (58.86%)	0.569784
Diabetes	10 (62.5%)	6 (37.5%)	72 (33.96%)	140 (66.04%)	0.021809	6 (37.5%)	10 (62.5%)	85 (40.09%)	127 (59.91%)	0.838092
Coronary heart disease	11 (57.89%)	8 (42.11%)	71 (33.97%)	138 (66.03%)	0.037484	5 (26.32%)	14 (73.68%)	86 (41.15%)	123 (58.85%)	0.206225
Arrhythmology	8 (36.36%)	14 (63.64%)	74 (35.92%)	132 (64.08%)	0.967297	8 (36.36%)	14 (63.64%)	83 (40.29%)	123 (59.71%)	0.720665
Cerebral infarction	12 (50%)	12 (50%)	70 (34.31%)	134 (65.69%)	0.12985	9 (37.5%)	15 (62.5%)	82 (40.2%)	122 (59.8%)	0.798633
Mental disease	0 (NaN %)	0 (NaN %)	82 (35.96%)	146 (64.04%)	1	0 (NaN %)	0 (NaN %)	91 (39.91%)	137 (60.09%)	1
Gastroesophageal reflux	18 (47.37%)	20 (52.63%)	64 (33.68%)	126 (66.32%)	0.108577	16 (42.11%)	22 (57.89%)	75 (39.47%)	115 (60.53%)	0.762353
Type II respiratory failure	48 (61.54%)	30 (38.46%)	34 (22.67%)	116 (77.33%)	6.54E‐09	24 (30.77%)	54 (69.23%)	67 (44.67%)	83 (55.33%)	0.042064
Budesonide/glycopyrronium/formoterol	30 (38.46%)	48 (61.54%)	49 (33.33%)	98 (66.67%)	0.443105	31 (39.74%)	47 (60.26%)	58 (39.46%)	89 (60.54%)	0.966484
Tiotropium	2 (7.14%)	26 (92.86%)	77 (39.09%)	120 (60.91%)	0.000921	13 (46.43%)	15 (53.57%)	76 (38.58%)	121 (61.42%)	0.426682
Salmeterol/fluticasone	3 (9.68%)	28 (90.32%)	76 (39.18%)	118 (60.82%)	0.001398	10 (32.26%)	21 (67.74%)	79 (40.72%)	115 (59.28%)	0.370853
Budesonide/formoterol	10 (22.73%)	34 (77.27%)	69 (38.12%)	112 (61.88%)	0.055012	22 (50%)	22 (50%)	67 (37.02%)	114 (62.98%)	0.11417
Hospitalized	NA	NA	NA	NA	NA	22 (26.83%)	60 (73.17%)	69 (47.26%)	77 (52.74%)	0.002502

Abbreviation: EOS, eosinophil.

We further investigated the association between EOSC and clinical outcomes in COPD patients infected with Omicron. By grouping patients based on EOSC (EOSC < 0.1 vs. EOSC ≥ 0.1), we found that a higher percentage of COPD patients with low EOSC required hospitalization after Omicron infection (73.17% vs. 52.74%, *p* < 0.01). Additionally, we observed that age (72.58 vs. 69.26, *p* = 0.02), WBC (7.82 vs. 7.09, *p* = 0.02), NEU (percentage: 66.4% vs. 63.27%, *p* = 0.02; count: 5.27 vs. 4.6, *p* = 0.01), CRP (27.64 vs. 20.15, *p* < 0.01) were higher in the low EOSC group, while LY (27.1% vs. 23.7%, *p* < 0.01), HB (143.78 vs. 137.37, *p* < 0.01), and ALB (39.1 vs. 36.99, *p* = 0.02) were higher in the high EOSC group. Type II RF was more prevalent in the low EOSC group (69.23% vs. 55.33%, *p* = 0.04). However, there was no significant difference in daily medication between the high and low EOSC groups. Moreover, we performed a correlation analysis between EOSC and other blood indices in 228 COPD patients infected with Omicron. The analysis revealed that EOSC was positively correlated with LY (Cor = 0.16, *p* = 0.02), and negatively correlated with age (Cor = −0.2, *p* < 0.01), NEU (percentage: Cor = −0.19, *p* < 0.01), and CRP (Cor = −0.27, *p* < 0.0001) (Tables 3 and 4, Figure 3C–E). The correlation analysis consistently demonstrated that COPD patients with high EOSC were generally less symptomatic and had lower hospitalization rates when infected with Omicron.

### 3.4 EOSC decreased in COPD patients after the Omicron infection

**FIGURE 4:**
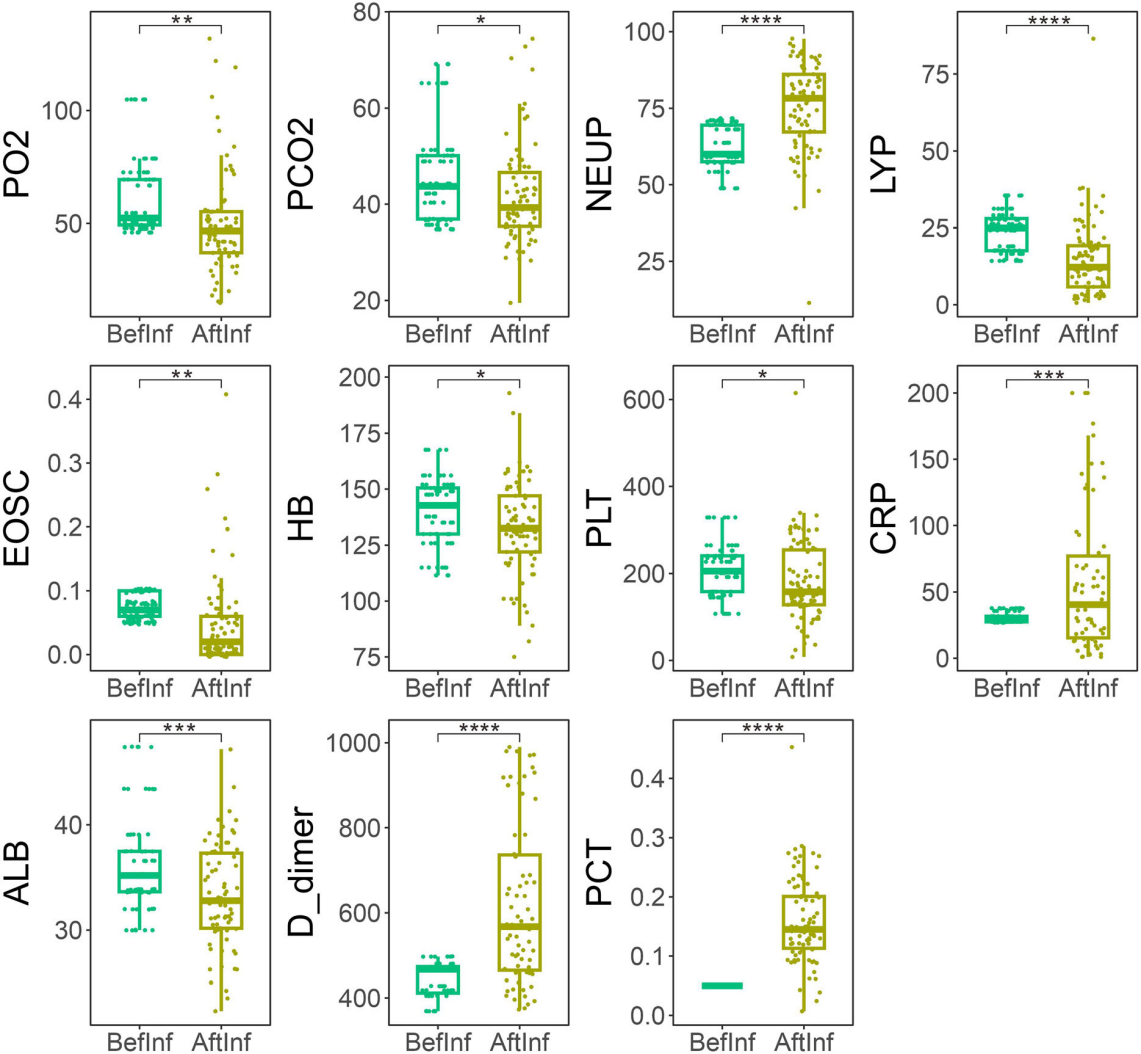
Blood indices were significantly different in chronic obstructive pulmonary disease patients before and after infection with Omicron.

After infection with Omicron, 82 COPD patients required hospitalization, and we conducted a follow‐up study to re‐measured blood indices. A comparison between the newly measured blood indices and those measured during the previous COPD treatment showed significant changes. We observed that PO_2_ (60.61 vs. 50.4, *p* < 0.01), PCO_2_ (46.01 vs. 41.96, *p* = 0.01), LY (23.36% vs. 14.49%, *p* < 0. 0001), EOS (count: 0.07 vs. 0.05, *p* < 0.01), HB (139.74 vs. 132.82, *p* = 0.02), PLT (209.35 vs. 181, *p* = 0.02), and ALB (36.33 vs. 33.27, *p* < 0.001) were all higher before infection than after Omicron infection. Conversely, NEU (percentage: 75.66% vs. 62.31%, *p* < 0. 0001), CRP (58.55 vs. 30.44, *p* < 0. 001), D_dimer (621.18 vs. 441.66, *p* < 0.0001), and PCT (0.16 vs. 0.05, *p* < 0.0001) were higher after the infection (Table 5, Figure 4). These results showed that EOS and LY decreased while CRP increased after Omicron infection, indicating inflammatory indicators increased.

**TABLE 5:** *T*‐test results of chronic obstructive pulmonary disease patients before and after Omicron infection.

Type	Before		After		*p*
	Total	Mean	Total	Mean	
Oxygen partial pressure	82	60.61	77	50.40	0.001654
Partial pressure of carbon dioxide	82	46.01	77	41.96	0.014224
White blood cell	82	8.12	82	7.81	0.745375
Neutrophil percentage	82	62.31	82	75.66	1.83E‐11
Neutrophil count	82	5.15	82	5.63	0.263713
Lymphocyte percentage	82	23.36	82	14.49	6.80E‐08
Lymphocyte count	82	2.05	82	1.53	0.504915
Eosinophil percentage	82	0.98	81	0.79	0.158757
Eosinophil count	82	0.07	81	0.05	0.001089
Hemoglobin	76	139.74	78	132.82	0.021908
Platelet	76	209.35	82	181.00	0.022311
C‐reactive protein	82	30.44	62	58.55	0.000208
Fibrinogen	30	3.59	30	3.45	0.666766
Albumin	70	36.33	76	33.27	0.000143
D_dimer	47	441.66	74	621.18	1.83E‐11
Procalcitonin	18	0.05	82	0.16	1.91E‐22

### 3.5 Positive correlation between EOSC and T‐cell counts in COPD patients after Omicron infection

**FIGURE 5:**
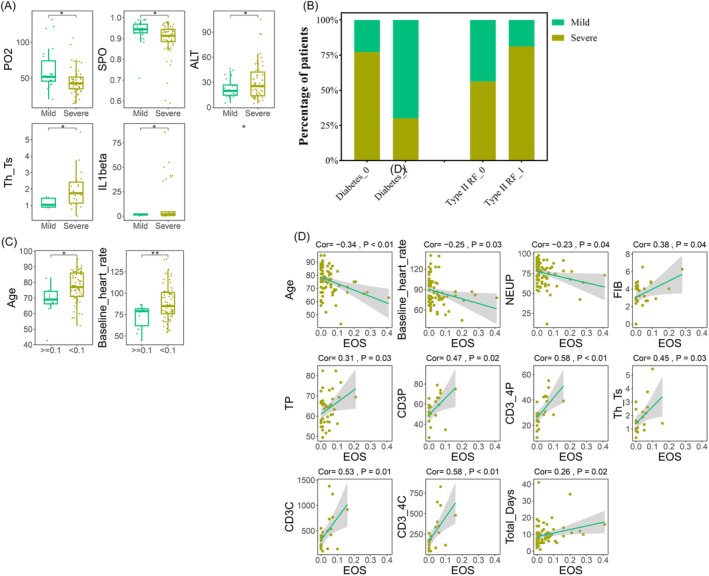
Clinical indicators of significant differences between mild and severe conditions in chronic obstructive pulmonary disease (COPD) patients hospitalized after infection with Omicron. (A) Blood indicators of significant differences between mild and severe condition patients; (B) co‐existing diseases and daily medication of significant differences between mild and severe condition patients; (C) blood indicators of significant differences between mild and severe condition patients grouped by eosinophil (EOS); (D) co‐existing diseases and daily medication of significant differences between mild and severe condition patients grouped by EOS; (E) blood indicators of significant differences between mild and severe condition patients grouped by EOS. (F) Blood indicators of significant differences between mild and severe condition patients grouped by EOS; (G) blood indicators of significant differences in EOS and daily medication of significant differences in mild and severe condition patients.

Out of the 82 hospitalized patients, 23 patients had a mild illness, 57 patients had a severe illness, and 2 patients were not included for missing detailed recorders. We compared the clinical characteristics between mild and severe illness patients and identified significant differences in nine indicators, of which PO_2_ (62.07 vs. 45.73, *p* = 0.02) and SPO_2_ (blood oxygen saturation) (0.94 vs. 0.9, *p* = 0.04) were higher in the mild illness group. ALT (alanine aminotransferase) (31.05 vs. 22.59, *p* = 0.03), Th_Ts (Th/Ts) (2 vs. 1.15, *p* = 0.01), and IL1beta (interferon‐1β) (12.47 vs. 1.4, *p* < 0.01) were higher in severe illness group. Additionally, we found that the presence of coexisting diseases was associated with disease severity. The proportion of patients without diabetes (77.14% vs. 30%, *p* < 0.01) and those with Type II RF (81.25% vs. 56.25%, *p* = 0.02) was higher in the severe illness group. In other coexisting diseases and daily medications, there were no significant difference between mild and severe illness groups (Tables 6 and 7, Figure 5A,B).

**TABLE 6:** *T*‐test results of chronic obstructive pulmonary disease patients infected with omicron between mild and severe hospitalization.

Type	SARS‐CoV‐2					EOS				
	Mild		Severe		*p*	EOS < 0.1		EOS > =0.1		*p*
	Total	Mean	Total	Mean		Total	Mean	Total	Mean	
Age	23	73.48	56	77.63	0.169226	70	77.39	10	68.20	0.006827
BMI	13	22.51	20	22.61	0.924937	30	22.66	3	21.66	0.582229
Baseline heart rate	23	86.52	56	87.73	0.809486	70	89.11	10	72.50	0.01209
Discharged heart rate	4	76.75	9	86.67	0.253455	13	NA	1	NA	NA
Baseline respiratory rate	23	20.83	57	21.53	0.525849	71	21.27	10	20.60	0.648542
Discharged respiratory rate	12	18.33	39	17.74	0.234847	44	17.98	7	17.29	0.258761
Blood pressure high	23	134.78	57	133.96	0.856055	71	135.31	10	130.60	0.454785
Blood pressure low	23	79.26	57	76.49	0.348339	71	76.85	10	80.50	0.363299
White blood cell	23	6.20	57	8.44	0.112231	71	7.85	10	6.83	0.435094
Neutrophil percentage	23	73.33	57	76.58	0.37281	71	75.97	10	71.43	0.360498
Neutrophil count	23	4.80	57	5.92	0.186538	71	5.59	10	5.07	0.646757
Lymphocyte percentage	23	15.73	57	14.08	0.593091	71	14.25	10	17.51	0.437666
Lymphocyte count	23	0.79	57	1.86	0.334448	71	1.62	10	1.06	0.535189
Monocyte percentage	23	10.03	57	8.54	0.210974	71	9.03	10	9.66	0.698822
Monocyte count	23	0.56	57	0.61	0.557389	71	0.60	10	0.61	0.949838
Eosinophil percentage	22	0.91	57	0.72	0.600188	71	0.50	10	2.84	0.000191
Eosinophil count	22	0.04	57	0.05	0.856047	71	0.02	10	0.20	0.00023
Fibrinogen	8	3.44	21	3.43	0.986239	27	3.35	3	4.41	0.274394
Red blood cell	23	4.33	57	4.12	0.220353	71	4.15	10	4.36	0.374684
Hemoglobin	20	139.30	56	130.77	0.060787	68	132.76	9	133.44	0.928795
Platelet	23	173.17	57	185.98	0.585569	71	173.48	10	236.00	0.239843
Partial pressure of carbon dioxide	22	43.85	55	41.21	0.314641	67	42.01	9	41.89	0.955968
Oxygen partial pressure	22	62.07	55	45.73	0.023441	67	50.42	9	49.63	0.925014
Blood oxygen saturation	23	0.94	55	0.90	0.041046	68	0.91	9	0.91	0.938773
Alanine aminotransferase	22	22.59	53	31.05	0.032832	66	28.43	9	29.08	0.927154
Aspartate transferase	2	NA	1	NA	NA	3	NA	0	NA	NA
Total protein	14	63.50	32	62.93	0.826535	42	62.64	4	67.95	0.206434
Albumin	23	34.66	52	32.74	0.115418	66	33.10	9	35.57	0.144646
Urea	22	6.55	53	7.39	0.462907	66	6.87	9	7.49	0.737788
Creatinine	23	86.29	55	89.91	0.586482	69	87.73	9	93.49	0.63366
D_dimer	20	551.40	52	649.23	0.05325	64	618.48	9	608.78	0.887219
C‐reactive protein	18	43.29	43	65.59	0.160569	54	58.74	8	57.28	0.945914
Erythrocyte sedimentation rate	11	28.73	29	35.48	0.379542	34	33.38	6	35.00	0.867026
Procalcitonin	13	0.65	40	0.38	0.358036	46	0.41	8	0.43	0.943699
B‐type natriuretic peptide	14	121.57	25	211.84	0.328241	33	195.39	6	74.83	0.076872
CD3+ percentage	5	50.32	18	56.07	0.447911	21	NA	2	NA	NA
CD3+/CD4+ percentage	5	25.10	18	32.66	0.187025	21	NA	2	NA	NA
Th/Ts	5	1.15	18	2.00	0.014301	21	NA	2	NA	NA
NK cell percentage	5	30.16	18	19.36	0.109782	21	NA	2	NA	NA
CD3+ CD19+ percentage	5	9.96	18	13.04	0.303636	21	NA	2	NA	NA
CD3+ count	5	372.00	18	479.89	0.55071	21	NA	2	NA	NA
CD3+ CD4+ count	5	185.40	18	280.94	0.137471	21	NA	2	NA	NA
Interleukin‐5	11	1.26	34	1.31	0.803086	38	1.29	7	1.29	0.984798
Interleukin‐4	12	0.70	38	1.70	0.053557	41	1.62	9	0.68	0.063785
Interleukin‐2	12	1.24	38	3.22	0.215112	41	3.18	9	1.15	0.181138
Interleukin‐10	12	10.71	38	8.09	0.608717	41	7.60	9	12.15	0.591719
Interferon‐α	11	44.78	34	2.63	0.191862	38	14.57	7	4.26	0.268116
Interferon‐1β	11	1.40	34	12.47	0.005439	38	11.28	7	7.65	0.664231
Interferon‐12P70	11	1.29	34	1.25	0.865655	38	1.25	7	1.15	0.794055
Interferon‐8	11	20.06	34	17.77	0.724367	38	16.12	7	20.50	0.528407
Interferon‐17A	3	3.08	8	6.25	0.509099	11	NA	1	NA	NA
Interferon‐6	12	48.15	38	100.56	0.328931	41	83.09	9	76.73	0.945799
Interferon‐γ	12	2.09	38	5.07	0.179845	41	4.84	9	3.59	0.626669
Tumor necrosis factor‐α	11	1.03	34	1.09	0.838408	38	1.03	7	1.28	0.621299
Hormone_Days	23	4.52	57	6.60	0.099343	71	5.87	10	6.30	0.833118
Prone_position_treatment_Days	14	8.57	44	9.50	0.55984	51	9.14	7	12.14	0.140768
Nidus	18	6.65	46	9.30	0.520582	56	7.95	9	7.24	0.890472
Ground_glass	18	0.06	45	1.43	0.289921	55	1.19	9	0.02	0.273171
Variation	18	6.58	44	8.27	0.660332	54	7.04	9	7.22	0.968892
Total_Days	23	9.04	57	9.67	0.736633	71	9.15	10	13.00	0.053623

Abbreviations: BMI, body mass index; EOS, eosinophil.

**TABLE 7:** Chi‐square/Fisher's test between mild and severe conditions in chronic obstructive pulmonary disease patients hospitalized after Omicron infection.

Type	State of an illness					EOS				
	Positive		Negative		*p*	Positive		Negative		*p*
	Severe	Mild	Severe	Mild		EOS ≥ 0.1	EOS < 0.1	EOS ≥ 0.1	EOS < 0.1	
Gender	16 (84.21%)	3 (15.79%)	41 (67.21%)	20 (32.79%)	0.152876	0 (0%)	19 (100%)	10 (16.13%)	52 (83.87%)	0.141228
Vaccine	23 (71.88%)	9 (28.12%)	14 (77.78%)	4 (22.22%)	0.903766	5 (15.15%)	28 (84.85%)	0 (0%)	19 (100%)	0.194895
Fungus	3 (100%)	0 (0%)	42 (72.41%)	16 (27.59%)	0.559878	0 (0%)	3 (100%)	9 (15%)	51 (85%)	1
Hypertension	23 (74.19%)	8 (25.81%)	34 (69.39%)	15 (30.61%)	0.643588	4 (12.9%)	27 (87.1%)	6 (12%)	44 (88%)	1
Diabetes	3 (30%)	7 (70%)	54 (77.14%)	16 (22.86%)	0.006776	1 (11.11%)	8 (88.89%)	9 (12.5%)	63 (87.5%)	1
Coronary heart disease	6 (60%)	4 (40%)	51 (72.86%)	19 (27.14%)	0.640617	0 (0%)	11 (100%)	10 (14.29%)	60 (85.71%)	0.397572
Arrhythmology	6 (75%)	2 (25%)	51 (70.83%)	21 (29.17%)	1	1 (12.5%)	7 (87.5%)	9 (12.33%)	64 (87.67%)	1
Chronic pulmonary heart disease	32 (71.11%)	13 (28.89%)	25 (71.43%)	10 (28.57%)	0.975172	6 (13.04%)	40 (86.96%)	4 (11.43%)	31 (88.57%)	1
Mental disease	0 (0%)	0 (0%)	57 (71.25%)	23 (28.75%)	1	0 (0%)	0 (0%)	10 (12.35%)	71 (87.65%)	1
Gastroesophageal reflux	12 (66.67%)	6 (33.33%)	45 (72.58%)	17 (27.42%)	0.625521	2 (11.76%)	15 (88.24%)	8 (12.5%)	56 (87.5%)	1
Nodules	16 (76.19%)	5 (23.81%)	41 (69.49%)	18 (30.51%)	0.560239	2 (9.52%)	19 (90.48%)	8 (13.33%)	52 (86.67%)	0.943107
Type II respiratory failure	39 (81.25%)	9 (18.75%)	18 (56.25%)	14 (43.75%)	0.015505	6 (12.77%)	41 (87.23%)	4 (11.76%)	30 (88.24%)	1
ARDS	0 (0%)	2 (100%)	57 (73.08%)	21 (26.92%)	0.080063	0 (0%)	1 (100%)	10 (12.5%)	70 (87.5%)	1
Cerebral Infarction	8 (66.67%)	4 (33.33%)	49 (72.06%)	19 (27.94%)	0.972406	1 (8.33%)	11 (91.67%)	9 (13.04%)	60 (86.96%)	1
Anemia	6 (85.71%)	1 (14.29%)	51 (69.86%)	22 (30.14%)	0.654124	0 (0%)	7 (100%)	10 (13.51%)	64 (86.49%)	0.588122
Renal Insufficiency	6 (85.71%)	1 (14.29%)	51 (69.86%)	22 (30.14%)	0.654124	1 (14.29%)	6 (85.71%)	9 (12.16%)	65 (87.84%)	1
Hypohepatia	3 (60%)	2 (40%)	54 (72%)	21 (28%)	0.949144	1 (20%)	4 (80%)	9 (11.84%)	67 (88.16%)	0.491838
Budesonide/glycopyrronium/formoterol	17 (60.71%)	11 (39.29%)	38 (77.55%)	11 (22.45%)	0.115669	4 (13.33%)	26 (86.67%)	6 (12.5%)	42 (87.5%)	1
Tiotropium	2 (100%)	0 (0%)	53 (70.67%)	22 (29.33%)	1	0 (0%)	2 (100%)	10 (13.16%)	66 (86.84%)	1
Salmeterol/fluticasone	3 (100%)	0 (0%)	52 (70.27%)	22 (29.73%)	0.553383	0 (0%)	3 (100%)	10 (13.33%)	65 (86.67%)	1
Budesonide/formoterol	10 (100%)	0 (0%)	45 (67.16%)	22 (32.84%)	0.076918	1 (10%)	9 (90%)	9 (13.24%)	59 (86.76%)	1
Hormone	50 (73.53%)	18 (26.47%)	7 (58.33%)	5 (41.67%)	0.467592	8 (11.76%)	60 (88.24%)	2 (15.38%)	11 (84.62%)	1
Antiviral	39 (72.22%)	15 (27.78%)	18 (69.23%)	8 (30.77%)	0.781864	5 (9.09%)	50 (90.91%)	5 (19.23%)	21 (80.77%)	0.35062
Baricitinib	6 (85.71%)	1 (14.29%)	51 (69.86%)	22 (30.14%)	0.654124	1 (14.29%)	6 (85.71%)	9 (12.16%)	65 (87.84%)	1
Glucocorticoid	43 (75.44%)	14 (24.56%)	14 (60.87%)	9 (39.13%)	0.192543	9 (15.79%)	48 (84.21%)	1 (4.17%)	23 (95.83%)	0.279185
Anticoagulant therapy	36 (73.47%)	13 (26.53%)	21 (67.74%)	10 (32.26%)	0.581344	6 (12.24%)	43 (87.76%)	4 (12.5%)	28 (87.5%)	1
Prone position treatment	44 (74.58%)	15 (25.42%)	13 (61.9%)	8 (38.1%)	0.270543	8 (13.56%)	51 (86.44%)	2 (9.09%)	20 (90.91%)	0.869681
Oxygen therapy	49 (70%)	21 (30%)	5 (71.43%)	2 (28.57%)	1	8 (11.59%)	61 (88.41%)	1 (12.5%)	7 (87.5%)	1
White lung	45 (71.43%)	18 (28.57%)	8 (66.67%)	4 (33.33%)	1	9 (14.06%)	55 (85.94%)	0 (0%)	12 (100%)	0.369859
State of an illness	NA	NA	NA	NA	NA	6 (10.53%)	51 (89.47%)	3 (13.64%)	19 (86.36%)	1

Abbreviation: EOS, eosinophil.

The statistical results after grouping by EOSC revealed significant differences in two indicators, including age (77.39 vs. 68.2, *p* < 0.01) and baseline heart rate (89.11 vs. 72.5, *p* = 0.01), which were all higher in the low EOSC group. Coexisting diseases and daily medications were no significant difference between the high and low EOSC groups. We also conducted a correlation analysis between EOS and other indicators in 82 hospitalized patients, and the results showed that age (Cor = −0.34, *p* < 0.01), baseline heart rate (Cor = −0.25, *p* = 0.03), and NEUP (Cor = −0.23, *p* = 0.04) were negatively correlated with EOSC. FIB (Cor = 0.38, *p* = 0.04), TP (total protein) (Cor = 0.31, *p* = 0.03), CD3P (CD3 + percentage) (Cor = 0.47, *p* = 0.02), CD3_4P (CD3+/CD4 + percentage) (Cor = 0.58, *p* < 0.01), Th_Ts (Th/Ts) (Cor = 0.45, *p* = 0.03), CD3C (CD3 + Count) (Cor = 0.53, *p* = 0.01), CD3_4C (CD3 + CD4 + count) (Cor = 0.58, *p* < 0.01), and Total_Days (Cor = 0.26, *p* = 0.02) were positively correlated with EOSC (Tables 6 and 7, Figure 5C,D). Our results indicated that T‐cell counts in hospitalized patients were positively correlated with EOSC whereas age and NEU maintain a negative correlation with EOSC.

## 4 DISCUSSIONS

The present study aimed to investigate the clinical indicators and outcomes of COPD patients during the Omicron outbreak at Shanxi Bethune Hospital. The clinical data from 315 patients allowed us to gain valuable insights into the infection rate, hospitalization rate, cure rate, and mortality rate among COPD patients during this period. Due to the hospital's capacity to receive patients, the number of patients included in the study was limited. During the Omicron outbreak, a surge in infections overwhelmed medical resources.^17^ Based on the collected information, we compared patients in groups based on infection, hospitalization, and EOS count to investigate the clinical differences. Our findings elucidated the impact of the pandemic on COPD patients and the possible roles of EOS in COPD patients against Omicron infection.

The rapid spread of the Omicron variant raised significant challenges for COPD patients due to the disruptions to healthcare services and the unknown comorbidities between COPD and the Omicron variant.^18^, ^19^ In this study, we observed that 80.32% of COPD patients were infected with Omicron during the outbreak. However, due to the lack of exact information, it remains unclear whether patients with COPD were at an increased risk of becoming infected with Omicron which should be involved in future analysis.^12^ Previous studies reported that COPD patients with consistently high EOS had a lower risk of mortality, while patients with fluctuating blood EOS had a higher risk of readmission,^20^ suggesting a positive role of EOS in the prognosis of COPD.^21^, ^22^, ^23^ In our study, we found that COPD patients with higher EOS levels were not only less likely to be infected with Omicron but also had a higher proportion of mild cases that did not require hospitalization, suggesting that EOSC may also serve as a potential indicator of protection against Omicron in COPD patients.^24^, ^25^, ^26^ This finding is consistent with previous studies showing that low EOS counts (<0.3 × 109 cells·L − 1) are associated with an increased risk of severe outcomes in COPD patients.^26^ Furthermore, we observed that EOS levels decreased after Omicron infection. Correlation analysis revealed a positive correlation between EOS levels and LY and T‐cells, and a negative correlation with NEU, CRP, and age. This suggests that older patients with lower EOS levels face a greater risk of Omicron infection.

T lymphocytes are also important determinants of disease stability in COPD, and individuals with stable disease have higher levels of IL‐2.^27^, ^28^ T‐cell‐mediated immune responses can alter the physiologic progression of COPD. In this study, we observed a positive correlation between EOSC and T‐cell counts in hospitalized patients after Omicron infection, indicating that EOSC might be associated with specific immune responses in COPD patients during viral infections and have an important effect on the clinical status of the patients. The low EOSC, which might also represent a low T2 immune status, was reported that associated with poor outcomes in asthmatic and possibly in non‐asthmatic COPD patients.^24^ Considering the positive correction, EOS counts could be used as an indicator of medical decisions regarding COPD patients during the Omicron outbreak, such as focusing on protecting patients with low EOS counts. Furthermore, COPD patients treated with BGF or BFF daily were more likely to be not infected with Omicron, providing a potential priority for using BGF and BFF in COPD patients.

In conclusion, our study provides valuable insights into the clinical indicators and outcomes of COPD patients during the Omicron outbreak. Our findings highlight EOS counts as potential indicators of protection against Omicron in COPD patients and offer valuable insights into the clinical characteristics and outcomes of COPD patients during the pandemic. However, the sample size may have influenced the statistical result. Future studies with larger and more diverse cohorts are needed to validate our findings and explore further the underlying mechanisms of the EOSC after the Omicron infection of COPD patients.

## 5 CONCLUSIONS

In summary, our study followed up with COPD patients during the Omicron outbreak at Shanxi Bethune Hospital between 1 September 2021 to 1 September 2022, including a total of 315 patients. Among all patients, 87 patients were not infected with Omicron, and 228 were infected with Omicron. The infected patients were further divided into 146 patients who did not require hospitalization and 82 patients who needed hospitalization. We found that COPD patients with high EOS levels were not only less likely to be infected with Omicron but also had a higher proportion of mild cases that did not require hospitalization, and EOS levels decreased after Omicron infection. Correlation analysis revealed a positive correlation between EOS levels and LY and T‐cells, and a negative correlation between EOS levels and age, NEU, and CRP.

## AUTHOR CONTRIBUTIONS

Ruiying Wang had the idea for and designed the study. Xiansheng Liu and Shuang Wei supervised the study. Xueli Bai, Yanan Niu, Zhifan Zhu, Min Xu, and Hu Liu did the statistical analysis. All authors contributed to acquisition, analysis, or interpretation of data. Xueli Bai wrote the draft report. All authors revised the report and approved the final version before submission.

## CONFLICT OF INTEREST STATEMENT

The authors declare that there is no conflict of commercial interest related to this paper.

## ETHICS STATEMENT

The present study was approved by the ethics committee of Shanxi Bethune Hospital ([2022] S022), and all participants provided written informed consent.

## Data Availability

Data sharing not applicable to this article as no datasets were generated or analysed during the current study.
